# CNP, the Third Natriuretic Peptide: Its Biology and Significance to the Cardiovascular System

**DOI:** 10.3390/biology11070986

**Published:** 2022-06-29

**Authors:** Yasuaki Nakagawa, Toshio Nishikimi

**Affiliations:** 1 Department of Cardiovascular Medicine, Kyoto University Graduate School of Medicine, 54 Shogoin-Kawara-cho, Sakyo-ku, Kyoto 606-8507, Japan; nishikim@kuhp.kyoto-u.ac.jp; 2 Department of Medicine, Wakakusa Tatsuma Rehabilitation Hospital, 1580 Ooaza Tatsuma, Daito City 574-0012, Japan

**Keywords:** C-type natriuretic peptide, CNP, GC-B, NPR-C

## Abstract

**Simple Summary:**

CNP is the third natriuretic peptide to be isolated and is widely expressed in the central nervous system, osteochondral system, and vascular system. The receptor that is mainly targeted by CNP is GC-B, which differs from GC-A, the receptor targeted by the other two natriuretic peptides, ANP and BNP. Consequently, the actions of CNP differ somewhat from those of ANP and BNP. Research into the actions of CNP has shown that CNP attenuates cardiac remodeling in animal models of cardiac hypertrophy, myocardial infarction, and myocarditis. Studies examining CNP/GC-B signaling showed that it contributes to the prevention of cardiac stiffness. Endogenous CNP, perhaps acting in part through CNP/NPR-C signaling, contributes to the regulation of vascular function and blood pressure. CNP regulates vascular remodeling and angiogenesis via CNP/GC-B/CGK signaling. CNP attenuates interstitial fibrosis and fibrosis-related gene expression in pressure overload and myocardial infarction models. The clinical application of CNP as a therapeutic agent for cardiovascular diseases is anticipated.

**Abstract:**

The natriuretic peptide family consists of three biologically active peptides: ANP, BNP, and CNP. CNP is more widely expressed than the other two peptides, with significant levels in the central nervous system, osteochondral system, and vascular system. The receptor that is mainly targeted by CNP is GC-B, which differs from GC-A, the receptor targeted by ANP and BNP. Consequently, the actions of CNP differ somewhat from those of ANP and BNP. CNP knockout leads to severe dwarfism, and there has been important research into the role of CNP in the osteochondral system. As a result, a CNP analog is now available for clinical use in patients with achondroplasia. In the cardiovascular system, CNP and its downstream signaling are involved in the regulatory mechanisms underlying myocardial remodeling, cardiac function, vascular tone, angiogenesis, and fibrosis, among others. This review focuses on the roles of CNP in the cardiovascular system and considers its potential for clinical application in the treatment of cardiovascular diseases.

## 1. Introduction

The natriuretic peptide family consists of three bioactive peptides: atrial natriuretic peptide (ANP), B-type natriuretic peptide (BNP), and C-type natriuretic peptide (CNP). ANP was first isolated and identified in 1983–1984 from rat and human cardiac atria [1,2]. BNP was subsequently isolated from the porcine brain in 1988, and CNP was also isolated from the porcine brain in 1990 [3,4]. The three peptides have similar structures and exert their biological actions, which include natriuresis and vasodilatation, by binding to specific receptors on the target organs. ANP and BNP are mainly synthesized in the heart. Currently, ANP is widely used in the treatment of acute heart failure in Japan [5,6,7], while BNP is used as a biomarker of heart failure worldwide [8,9,10]. CNP, by contrast, is widely expressed in the central nervous system, osteochondral system, and vascular system, and the receptor mainly targeted by CNP differs from that targeted by ANP and BNP. Consequently, the actions of CNP differ from those of ANP and BNP. As global CNP knockout mice (CNP KO) reportedly showed severe dwarfism, due to the impairment of endochondral ossification [11], research into CNPs has been preceded by studies on their role in the osteochondral system. In fact, the CNP analog has been developed for clinical application as a drug for achondroplasia [12]. In this review, we focus on CNP and review its biology and physiology, its significance in the pathophysiology of cardiovascular disease, and its therapeutic potential.

The CNP gene (*Nppc*) is used as a template for the transcription of PreproCNP. The N-terminal signal peptide is cleaved from PreproCNP, yielding proCNP, which is processed to form NT-proCNP, CNP-53, and CNP-22. CNP-53 is generated from proCNP by furin, but the enzyme catalyzing the generation of CNP-22 is still unknown. Both CNP-53 and CNP-22 are biologically active and bind to both NRP-B (GC-B) and NRP-C.

## 2. Structure and Physiology of CNP

**Figure 1:**
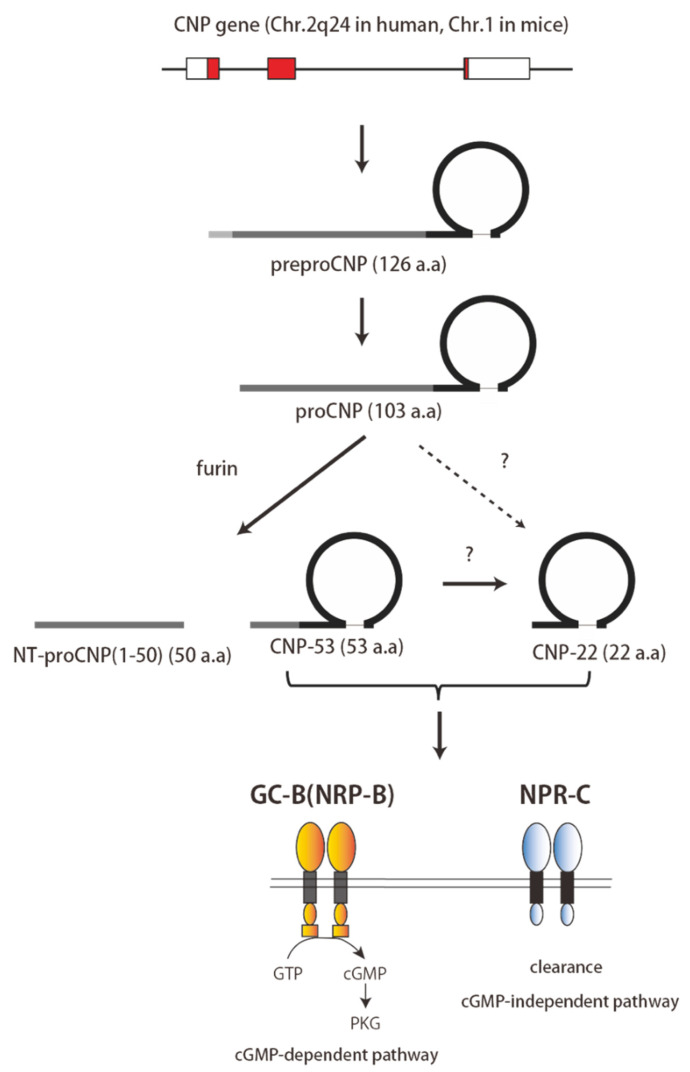
Schematic representation of CNP production and its receptors.

**Figure 2:**
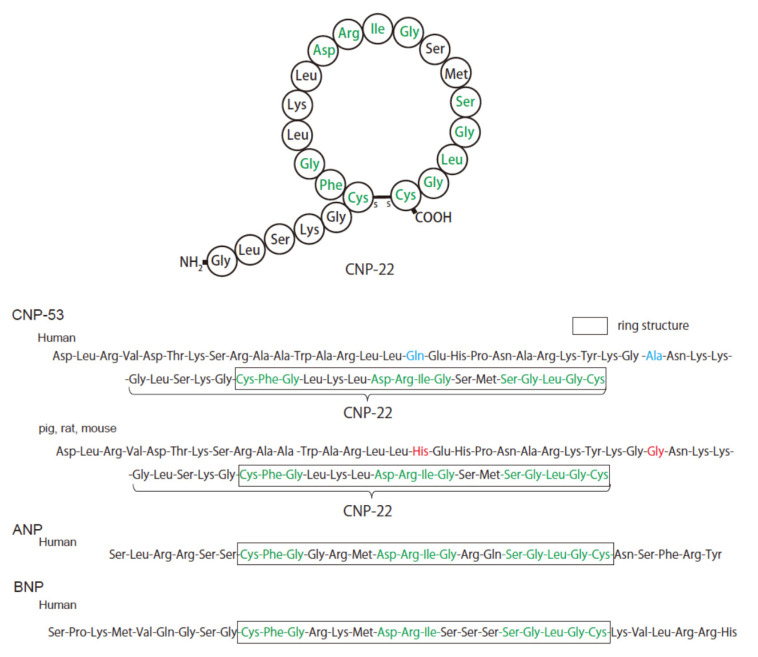
Schematic representation of the amino acid sequence of CNP. The upper panel shows a schema of the structure and amino acid sequence of CNP-22. The lower panels show the amino acid sequences of CNP-53 and CNP-22 in human, mouse, rat, and pig models in comparison with those of human ANP and BNP. Red- and blue-colored characters depict the amino acid residues that differ between human CNP and pig, mouse, or rat CNP. Green-colored characters depict the amino acid residues that are common among the three types of natriuretic peptides. Boxes indicate the amino acid residues comprising the ring structures.

CNP is translated as a 126-amino-acid prepropeptide and is then processed by a signal peptidase into ProCNP, a peptide of 103 residues (Figure 1). ProCNP is thought to subsequently be cleaved by the processing enzyme, furin, into NT-proCNP and CNP-53 [13,14]. CNP-53 is then cleaved by an unknown protease to produce CNP-22 [15,16]. Both CNP-53 and CNP-22 are present in plasma and various tissues, where both exhibit biological activity [17] (Figure 1). CNP-22 is highly homologous to ANP and BNP and is composed of 22 amino acids with a conserved disulfide-linked 17-amino acid ring portion that is required for biological activity [18] (Figure 2). However, CNP lacks a C-terminal tail and has a cysteine residue at the C-terminus, which is different from ANP and BNP. CNP-53 has a 31-amino-acid N-terminal extension that cleaves to form CNP-22 [18] (Figure 2). CNP is highly conserved across species. The amino acid sequence of CNP-22 is identical in human, pig, rat, and mouse peptides, while human CNP-53 differs from the pig, rat, and mouse peptides by only two amino acids [19,20] (Figure 2). CNP is expressed in a variety of central and peripheral tissues, including the brain, chondrocytes, and vascular endothelium [14]. In the heart, its expression has also been documented in the myocardium and cardiac fibroblasts [14]. 

*Nppc*, the gene encoding CNP, is located on chromosome 2q24 in humans and chromosome 1 in mice [14,18]. On the other hand, both the *Nppa*, encoding for ANP, and *Nppb*, encoding for BNP, are located on chromosome 1 in humans and chromosome 4 in mice. Because *Nppc* is physically separated from *Nppa* and *Nppb*, CNP is thought to be functionally and evolutionarily distinct from ANP and BNP [18].

There are three natriuretic peptide receptors: guanylyl cyclase-A (GC-A) (or natriuretic peptide receptor-A (NPR-A)), guanylyl cyclase-B (GC-B) (or natriuretic peptide receptor-B (NPR-B)), and natriuretic peptide receptor-C (NPR-C). GC-A is the main receptor for ANP and BNP and mediates their physiological action by generating the intracellular second messenger cyclic guanosine-3′,5′-monophosphate (cGMP). GC-B is the main receptor for CNP. Upon intravenous administration, CNP elicits a smaller increase in plasma cGMP levels than does ANP, suggesting that GC-B is less available to intravenously injected CNP than GC-A is to ANP [18]. NPR-C is not linked to guanylyl cyclase and appears to act, in large part, to clear natriuretic peptides from the circulation. The affinity of the natriuretic peptides for their receptors is as follows; GC-A, ANP > BNP >> CNP; GC-B, CNP >> ANP > BNP; NPR-C, ANP > CNP = BNP [14].

CNP has similar binding affinities to GC-B and NPR-C, although the signaling pathways activated by the two receptors are remarkably different. GC-B catalyzes the conversion of guanosine-5′-triphosphate to cGMP, which is a second messenger that initiates the activation of the protein kinases G-I and G-II (PKG-1 and PKG-II). GC-B is predominantly expressed in the brain [14] but is also found in kidney, lung, adrenal, uterus, and ovarian tissue [14]. Within the cardiovascular system, GC-B is co-expressed with GC-A in nearly all cell types [21]. 

NPR-C is mainly expressed in the kidney, lungs, and vasculature, and at low levels in all other organs [21]. NPR-C is composed of 540 amino acids. It does not contain a guanylyl cyclase domain and was initially thought to be a clearance receptor with no signaling activity [14]. However, several studies have shown that NPR-C mediates the effects of CNP, independently of cGMP. The mature NPR-C protein forms a homodimer that is stabilized by intermolecular disulfide bonds. The intracellular region of each subunit is composed of 37 amino acids, forming overlapping G_i/o_ binding sequences. Thus, in vitro studies suggest that the binding of natriuretic peptides to NPR-C can be the trigger for a G protein-linked process via a G_i_-binding domain at its intracellular C-terminus. This includes the inhibition of adenylyl cyclase activity (by the G_i_ α subunit) and the activation of phospholipase-Cb3 turnover (by the G_i_ βγ subunit) [22]. In addition, it was reported that NPR-C mediates the vascular actions of endothelial CNP, as described later in this review. 

In addition to clearance via NPR-C, natriuretic peptides are also inactivated via cleavage by neprilysin. This protease cleaves ANP and CNP at the ring and at other sites, resulting in their inactivation. Notably, neprilysin hydrolyzes CNP even more rapidly than ANP [23]. By contrast, BNP is comparatively resistant to neprilysin-catalyzed degradation in humans [24]. 

## 3. Distribution of CNP and Its Receptor in the Heart

The presence of CNP in the heart has been demonstrated using radioimmunoassays, immunohistochemistry, and RT-PCR [25]. Using immunohistochemistry, for example, CNP was detected in cardiac tissue sections and H9c2 cells from a rat cardiomyoblast cell line [25]. On the other hand, using HPLC, Horio et al. [26] found that cardiomyocytes do not express CNP mRNA, whereas fibroblasts do so. Using a CNP radioimmunoassay in combination with HPLC, they also observed that fibroblasts secrete CNP. The secretion of CNP by the heart is described in detail below.

The distribution of receptor expression and the effect of CNP in target cells, including cardiac myocytes and fibroblasts, have been described in several reports. ANP and BNP (GC-A agonists) both induced cGMP generation in purified rat ventricular myocytes, whereas CNP (a GC-B agonist) had no effect [27]. This suggests that rat ventricular myocytes predominantly express GC-A. However, Tokudome et al. detected the significant expression of both GC-A and GC-B in cultured rat cardiomyocytes, with greater expression of GC-B. Moreover, CNP increased the cellular cGMP levels in cardiomyocytes to a greater extent than did ANP, and CNP dose-dependently decreased the incorporation of [^3^H] leucine into cardiomyocytes under unstimulated conditions, whereas ANP did not do so [28]. Increased CNP also attenuated the Ca^2+^ influx, mediated by endothelin-1 (ET-1), the activation of calmodulin-dependent protein kinase II, ERK phosphorylation, and the DNA binding activity of the transcription factors GATA-4 and myocyte enhancer factor-2 (MEF-2) in cultured cardiomyocytes [28]. Fujisaki et al. reported that both cardiomyocytes and cardiac fibroblasts express natriuretic peptide receptor mRNA, that fibroblasts express both GC-A and GC-B, and that ANP, BNP, and CNP similarly increased the intracellular cGMP levels in cardiac fibroblasts [29]. Consistent with those findings, Cao and Gardiner reported that GC-A, GC-B, and NPR-C mRNA are all expressed in cardiac fibroblasts and that ANP, BNP, and CNP increase intracellular cGMP levels [30]. They also observed in the same study that all three peptides suppress the DNA synthesis induced by angiotensin II, ET-1, acidic and basic fibroblast growth factor, and insulin-like growth factor I. Tokudome et al. also reported that CNP and 8-bromo cGMP attenuated the ET-1 secretion from cardiac fibroblasts [28]. These observations suggest that CNP, like ANP and BNP, potentiates antihypertrophic and anti-fibrotic effects in the heart.

## 4. Role of CNP in the Pathophysiology of the Heart

Several in vivo models have been used to evaluate the effects of CNP. Using a sub-pressor angiotensin II-induced hypertrophy model in mice, Izumiya et al. showed that the infusion of CNP (0.05 mg/kg/min), conducted simultaneously with an angiotensin II treatment for 2 weeks, significantly attenuated the angiotensin II-induced increase in left ventricular wall thickness, chamber dilatation, and the decrease in fractional shortening [31]. In addition, CNP reduced angiotensin II-induced increases in cardiomyocyte size and suppressed hypertrophic-related gene expression, including the expression of ANP, BNP, and the β-myosin heavy chain [31]. These cardioprotective effects occurred without lowering systemic blood pressure. Soeki et al. examined the effects of CNP in a rat myocardial infarction model [32]. Administration of CNP at 0.1 mg/kg/min markedly attenuated the left ventricular remodeling and improved hemodynamics following the induction of myocardial infarction by coronary artery ligation in Sprague-Dawley rats. CNP significantly reduced the cross-sectional area of cardiomyocytes and reduced the transcription of cardiac remodeling-related genes in non-infarct regions. In addition, Obata et al. reported that CNP administration significantly improved the left ventricular systolic function and hemodynamics in a rat myocarditis model [33]. CNP also attenuated myocarditis-induced necrosis and infiltration by inflammatory cells [33]. Again, all these cardioprotective effects of CNP occurred without affecting arterial blood pressure. Wang et al. used transgenic (TG) mice that were overexpressing CNP in cardiomyocytes, to assess the effects of CNP in ischemia-reperfusion (I/R) and myocardial infarction [34]. They found that in the I/R model, the infarct size did not differ between wild-type and TG mice. In the myocardial infarction model, the left and right ventricular hypertrophy was attenuated in TG mice for 3 weeks post-infarction. There was also less post-infarction necrosis, muscular degeneration, and inflammation in the TG mice [34]. Langenickel et al. evaluated TG rats that were systemically overexpressing a dominant-negative GC-B mutant, which selectively reduced GC-B signaling without affecting GC-A signaling [35]. These rats showed progressive cardiac hypertrophy without fibrosis, as well as reduced bone growth and a moderate increase in heart rate. On the other hand, there were no significant differences in systolic, diastolic, or mean arterial pressure between TG and wild-type rats. These in vivo findings suggest that CNP/GC-B signaling exerts protective effects in the heart under various pathological conditions. 

Recently, Michel et al. reported that by stimulating GC-B and cGMP/cGMP-dependent kinase 1 (CGK1) signaling, CNP exerted potent protective effects in terms of heart failure, with a preserved ejection fraction (HFpEF) [36]. HFpEF is associated with high morbidity and mortality, comparable to heart failure, with a reduced ejection fraction (HFrEF) [37]. Although the development of new therapeutic agents has increased the treatment options for patients with HfrEF, no specific treatment for HfpEF has yet been developed. Consequently, there is an urgent need for the development of a therapeutic agent for the treatment of HFpEF. Titin is known to be associated with the stiffness of cardiomyocytes and, therefore, with overall myocardial wall tensibility and diastolic function [38]. Titin is modulated by posttranslational modification, including the oxidation and phosphorylation of its spring segment, while the phosphorylation of specific residues within titin by CGK1 decreases cardiomyocyte stiffness (38). CGK1 activity and phosphorylation were markedly reduced in tissues from patients with heart failure, in whom the passive myocyte stiffness was increased [39]. Using a transverse aortic constriction (TAC) model in mice, Kuhn et al. demonstrated that titin phosphorylation was enhanced in the model mice. In addition, cardiomyocyte-specific GC-B KO mice that were subjected to TAC showed mild ventricular dysfunction, together with cardiomyocyte stiffness, which effects were not seen in wild-type mice [36]. In addition, recombinant CGK1 reduced titin phosphorylation and ameliorated the passive stiffness of GC-B-deficient cardiomyocytes in vitro [36]. Taken together, these observations suggest that the activation of GC-B/CGK1 pathways induced in cardiomyocytes by CNP provides protection against the increases in cardiac stiffness induced by pressure overload and may, thus, be effective against myocardial stiffness in HFpEF.

Accumulating evidence suggests that heart rate (HR) is an important factor affecting cardiac performance and prognosis in patients with heart failure. While severe bradycardia results in low cardiac output and heart failure, sustained tachycardia worsens the prognoses of patients with heart failure. Large clinical trials have shown that lowering HR using a hyperpolarization-activated cation (HCN) channel inhibitor and/or a beta-blocker improves the prognosis of heart failure patients. Therefore, maintaining an optimal heart rate is very important. Recently, Dorey et al. evaluated HR regulation and sinoatrial node (SAN) function in heterozygous GC-B KO mice, which exhibit a slow HR, increased corrected SAN recovery time, and slowed SAN conduction, without evidence of cardiac hypertrophy or a change in cardiac function [40]. Hyperpolarization activated currents (*I*_f_) and L-type Ca^2+^ currents (*I*_CaL_) were reduced due to lower levels of cGMP and the increased hydrolysis of cAMP by phosphodiesterase III (PDE III) in the SAN. The application of 8-Br-cGMP, an analog of cGMP, restored the *I*_f_ and *I*_CaL_ as well as SAN conduction. These data suggest that GC-B signaling is involved in the maintaining of normal HR and SAN function via the cGMP/PDE III/cAMP signaling mechanism [40].

## 5. Role of CNP in the Regulation of Vascular Tone and Blood Pressure

CNP is mainly expressed by endothelial cells, whereas GC-B is co-expressed with GC-A in nearly all cell types within the cardiovascular system [41]. Within the vascular wall, high concentrations of GC-B are detected in endothelial cells, as well as the adjacent smooth muscle cells and fibroblasts [42]. CNP induces vasodilatation, not only by stimulating GC-B/cGMP but also by activating G_i_-coupled NPR-C receptors, which leads to the hyperpolarization of vascular smooth muscle cells [43]. However, the vasodilatation and reduction of blood pressure induced by CNP are much smaller than those elicited by ANP [44]. For that reason, it had previously been thought that the CNP/GC-B system is less important than ANP/GC-A for the regulation of vascular tone. However, we recently demonstrated that endogenous endothelial-derived CNP contributes to chronic blood pressure regulation in vivo [45]. As mentioned above, systemic CNP KO leads to early death due to osteochondral abnormality. We therefore generated and analyzed endothelial-specific CNP KO (CNP ecKO) and vascular smooth muscle cell-specific GC-B KO (GC-B smcKO) mice. These mice did not exhibit the skeletal abnormality or early death seen in systemic CNP or GC-B KO mice. Instead, CNP ecKO mice showed significantly increased systemic blood pressure and the enhanced acute response of blood pressure elevation to nitric oxide synthetase inhibition. In addition, acetylcholine-induced and endothelium-dependent vasorelaxation were impaired in rings of the mesenteric artery from CNP ecKO mice. By contrast, blood pressures in GC-B smcKO mice were similar to those in control mice, and the acetylcholine-induced vasorelaxation of mesenteric arterial rings was preserved. However, CNP-induced acute vasorelaxation was almost completely abolished in GC-B smcKO mice. These results suggest that endothelial-derived CNP contributes to the chronic regulation of vascular tone and systemic blood pressure, independently of GC-B, in the vascular smooth muscle [45]. 

Other groups have also reported the significance of CNP in the regulation of vascular tone and blood pressure. Moyes et al. reported that whereas female CNP ecKO mice show endothelial dysfunction and hypertension, the males do not. Moreover, ApoE KO mice showed a greater formation of atherosclerotic plaque and aneurysms than ApoE KO mice crossed with CNP ecKO mice [46]. They also detected endothelial dysfunction in the mesenteric arteries and a hypertensive phenotype in female NPR-C KO mice, but not in the males. In addition, exogenous CNP-induced hypotensive responses were diminished in NPR-C KO mice, suggesting that CNP/NPR-C signaling contributes to the maintenance of vascular homeostasis. Spiranec et al. reported that CNP-induced GC-B/cGMP signaling in microvascular smooth muscle cells and pericytes plays an essential role in the maintenance of normal microvascular resistance and blood pressure [47]. These investigators evaluated endothelial cell-specific GC-B KO mice, as well as precapillary arteriolar smooth muscle cell- and capillary pericyte-specific GC-B KO mice. GC-B is normally expressed in both endothelial cells and pericytes. The vasodilatory effect of CNP was abolished in mice lacking GC-B in the microcirculatory smooth muscle and pericytes but was preserved in mice lacking GC-B in the endothelial cells. CNP-mediated GC-B/cGMP signaling leads to the activation of CGK1 and the inhibition of phosphodiesterase IIIA (PDE IIIA), which, in turn, leads to the phosphorylation of vasodilator-stimulated phosphoprotein at both Ser239 (the CGK1-specific) and Ser157 (the cAMP/PKA-specific site). All these effects were abolished in GC-B-lacking pericytes. In mice lacking GC-B in the microcirculatory smooth muscle cells and pericytes, both peripheral vascular resistance and elevated blood pressure were elevated [47]. Despite some differences, these results indicate that CNP contributes to the maintenance of blood pressure, although further investigation of the target receptors and cell types involved is needed. Until now, no therapeutic agents have targeted CNP-related signaling. However, as mentioned above, CNP is degraded by neprilysin and its affinity is rather higher than that of other natriuretic peptides, so inhibiting NEP may enhance the action of CNP. Indeed, it has been reported that the plasma concentration of CNP in healthy subjects increases when sacubitril/valsartan, which is an angiotensin receptor–neprilysin inhibitor (ARNI), is administered [48]. Recently, ARNI has been clinically applied for hypertensive patients and has actually shown a favorable blood-pressure-lowering effect [49]; CNP, as well as ANP, may be involved in this effect. In combination with the data from the basic research described above, it is possible that CNP-related signals could be applied as a drug for blood-pressure control.

## 6. Role of CNP in Vascular Remodeling and Angiogenesis

Yamahara et al. reported that CNP acts via CGK1 to play a key role in angiogenesis [50]. CGK1 KO mice that were subjected to hindlimb ischemia (HLI), a well-established ischemia model, showed significantly poorer limb perfusion than wild-type mice throughout the 28-day follow-up after the induction of ischemia. Capillary density in the ischemic limb was lower in the CGK1 KO mice than in CGK1-overexpressing transgenic mice. Moreover, the adenoviral vector-mediated CNP gene transfer ameliorated the hindlimb ischemia. They also found that CNP significantly enhanced capillary network formation in vitro and that a CGK1 inhibitor blocked that effect [50]. Using HLI-model mice, Bubb et al. observed that pronounced vascular remodeling activity to restore an adequate blood supply occurred over the 28 days following the induction of ischemia [51]. On day 3 after HLI, the expression of CNP and VEGF-A mRNAs was reduced in the ischemic tissues, while the expression of hypoxia-inducible factor 1a, Bax, VCAM-1, and interleukin-6 mRNAs was enhanced. On the other hand, in endothelial-specific CNP KO mice, the recovery of perfusion after HLI was significantly impaired, with reductions in both blood vessel density and muscle fiber regeneration. These phenotypes were reversed by the administration of CNP [51], suggesting that CNP exerts pro-angiogenesis effects in HLI models. Those investigators also reported the effect of endogenous endothelial CNP in a mouse carotid artery wire-injury model. Neointimal hyperplasia, induced in response to the wire injury, was enhanced in endothelial-specific CNP KO mice as compared to control mice. In addition, global NPR-C KO mice exhibited a similarly exacerbated response to wire injury.

## 7. Role of CNP in Fibrosis in Cardiovascular Disease

Horio et al. reported that cardiac fibroblasts from adult rats secrete immunoreactive CNP in response to several humoral factors, including transforming growth factor-β1, basic fibroblast growth factor, and endothelin-1 [26]. The expression of both CNP and GC-B mRNA has been detected in cardiac fibroblasts and, by stimulating cGMP production, CNP inhibited both DNA and collagen synthesis in cardiac fibroblasts to a greater extent than did ANP and BNP [26]. It also appears that NPR-C may be involved in the anti-fibrotic effect of CNP that is seen in cultured human cardiomyocytes [52]. The NPR-C antagonist cANF4-28 blocked the BNP-mediated inhibition of cardiac fibroblast proliferation, but the GC-A/B inhibitor HS-142-1 did not do so. Moreover, in a mouse angiotensin II infusion model, CNP reduced the angiotensin II-induced increases in interstitial fibrosis and suppressed fibrosis-related gene expression, including type I and type III collagen [31]. Soeki et al. reported that the continuous infusion of CNP for 2 weeks after myocardial infarction suppressed cardiac fibrosis and attenuated left ventricular dysfunction [32], while Sangralingham et al. found that circulating CNP declined during aging and that the effect was strongly negatively correlated with left ventricular levels of interstitial fibrosis in rats [53]. 

CNP also significantly attenuated bleomycin-induced pulmonary fibrosis in mice, as indicated by significant decreases in Ashcroft scores (a histological indicator of lung fibrosis) and lung hydroxyproline content [54]. Consistent with that finding, CNP significantly reduced the number of macrophages, neutrophils, and lymphocytes, as well as interleukin (IL)-1β levels in bronchoalveolar lavage fluid, suggesting the anti-fibrotic effect of CNP reflects, in part, an anti-inflammatory effect. In addition, CNP markedly reduced the numbers of Ki-67-positive cells within fibrotic lesions in the lung, which suggests that CNP may directly inhibit the proliferation of pulmonary fibroblasts [54].

## 8. Increased Plasma CNP Levels in Heart Failure

Given that the other two natriuretic peptides, ANP and BNP, are produced in the heart and are increased in heart failure, the relationship between CNP and heart failure has been well studied. Based on Northern blot analysis, Takahashi et al. reported an absence of significant levels of CNP mRNA in the ventricles of failing hearts [55]. Soon after, Wei et al. [56] used a radioimmunoassay (with the Peninsula antibody) to measure plasma CNP levels in heart failure patients. They found that the plasma levels of ANP and BNP were higher in heart failure patients than in healthy controls, but the CNP levels did not differ between the heart failure patients and the controls. On the other hand, using immunohistochemistry, they detected CNP in the myocardium of both the controls and the heart failure patients, and the levels were higher in the latter [56]. Because all three natriuretic peptides have a common 17-amino-acid ring structure and strong sequence homology, antibodies against CNP may cross-react with ANP or BNP. Consequently, when tissue or plasma concentrations of ANP or BNP are very high, measurements made using a CNP antibody must be interpreted cautiously, as even a slight cross-reaction with ANP or BNP could erroneously enhance what is thought to be CNP staining [57]. When Cargill et al. [58] measured plasma CNP levels in heart failure patients using a commercial radioimmunoassay (Peninsula), they detected no increase in plasma CNP levels, compared to controls. Likewise, Hama et al. [59] used a CNP radioimmunoassay developed in-house and found that plasma CNP levels were not increased in heart failure patients, compared to controls. On the other hand, they showed that plasma CNP levels were elevated compared to controls in patients with cor pulmonare, sepsis, or renal failure [58,59].

Within the tissues, ProCNP1-103, a CNP precursor, is processed by furin into N-terminal proCNP1-50 (NT-proCNP) and CNP51-103 (CNP-53). CNP-53 is then further processed by an unknown enzyme into CNP82-103 (CNP-22) and CNP51-81, after which both are secreted into the blood. Prickett et al. measured plasma NT-proCNP levels in heart failure patients using an NT-proCNP radioimmunoassay developed in-house, in combination with size-exclusion HPLC and showed that NT-proCNP levels were higher in heart failure patients than in controls [60]. Importantly, the NT-proCNP antibody did not cross-react with N-terminal proANP or N-terminal proBNP, due to the low homology among the three N-terminal peptides. Moreover, the longer half-life of NT-proCNP makes it more detectable than CNP. The same group also investigated the potential for plasma NT-proCNP to serve as a marker of heart failure in symptomatic patients with recent-onset dyspnea and/or peripheral edema. They measured plasma NT-proCNP and other plasma vasoactive hormones, as well as echocardiographic indices. They found that plasma NT-proCNP is elevated in heart failure patients, compared with controls, and that plasma NT-proCNP levels correlate with the levels of other hormones but not with echocardiographic indices [61]. Karla et al. [62] measured plasma CNP and BNP levels in the coronary sinus and aorta in heart failure patients and showed that there is a stepping-up of plasma CNP levels in the coronary sinus compared to the aorta, indicating the production of CNP in the failing heart. However, the increase in plasma CNP (+29%, 1.04 pg/mL) is substantially smaller than the increase in plasma BNP (+57%, 49 pg/mL). The finding that plasma CNP levels were higher in the coronary sinus than in the aorta was replicated in a later study, although it remains unclear which cells in the heart secrete CNP [63,64]. Horio et al. [26] examined the expression of CNP and GC-B mRNA in cultured cardiomyocytes and fibroblasts. They found that fibroblasts express both CNP and GC-B mRNA, whereas cardiomyocytes express only NPR-B mRNA. They then used a CNP radioimmunoassay, combined with HPLC, to analyze a culture medium conditioned by fibroblasts and found that fibroblasts secrete authentic CNP, as the elution time was the same as that of CNP-22. Collectively, these results suggest that, unlike ANP and BNP, CNP is not a cardiac hormone that is produced and secreted by cardiomyocytes. 

## 9. The Source and Clinical Significance of CNP in Heart Failure

To investigate CNP production in organs other than the heart, Charles et al. [65] carried out trans-organ arteriovenous sampling in the liver, heart, hindlimb, and kidney in both control and heart failure sheep and measured CNP, NT-proCNP, ANP, and BNP. They found that ANP and BNP levels were significantly higher on the venous side of the heart than on the arterial side and that ANP and BNP levels were lower on the venous side in all other organs tested. This indicates that ANP and BNP are secreted from the heart, while the other organs are targets of the two peptides. By contrast, CNP and NT-CNP levels were both slightly higher on the venous side of the liver, heart, hindlimb, and kidney than on the arterial side, suggesting the production of CNP and NT-CNP in all these organs. That finding was consistent with an earlier study, in which arteriovenous samples were collected from various organs to measure adrenomedullin [66]. Adrenomedullin is highly expressed in vascular endothelial and smooth muscle cells, and the source of plasma adrenomedullin is thought to be the vascular wall. Therefore, the results with CNP and NT-proCNP are consistent with the hypothesis that the source of plasma CNP is the endothelium. The levels of both CNP and adrenomedullin are increased in proportion to the severity of heart failure; however, the magnitude of that increase is modest, and plasma CNP and adrenomedullin often only modestly correlate with measures of cardiac function, such as LVEF [61,67]. Furthermore, because both CNP and adrenomedullin mRNA are strongly induced in vascular endothelial cells by cytokines, such as tumor necrosis factor-α or interleukin-1β [68,69], increased plasma CNP levels may reflect a chronic inflammatory state in heart failure [70]. The aforementioned observation that CNP or NT-proCNP is modestly elevated in heart failure and often correlates modestly with the indices of cardiac function was replicated in later studies [71,72,73]. In addition, with exercise therapy, plasma CNP levels reportedly decreased along with BNP levels and symptoms [74]. Lok et al. investigated the prognostic power of the NT-proCNP in patients with chronic heart failure and found that NT-proCNP is a strong independent marker of outcome in patients with HFpEF, but not in those with HFrEF [75]. Among patients treated with sacubitril/valsartan, it was observed that those with baseline CNP levels below the median exhibited an overall increase in CNP levels at the end of the study, whereas those with CNP levels above the median at baseline exhibited a decrease in CNP levels at the end of the study [76].

Thus, with advances in detection methods, it has become clear that low concentrations of CNP and NT-proCNP, mainly of vascular endothelial origin, are present in the blood and are increased in proportion to the severity of heart failure, but the change is modest. In addition, a more recent study using a genetically engineered mouse model has shown that a small amount of cardiac-derived CNP may affect cardiac structure and function in heart failure [77]. The exact cause of the increased CNP in heart failure is still unknown, but it may reflect the inflammatory status and may be a useful prognostic indicator in patients with HFpEF. Further study will be needed to elucidate the exact meaning of the increased plasma CNP that is seen in heart failure.

## 10. Role of CNP in Pathogenesis in Pulmonary Arterial Hypertension

Pulmonary arterial hypertension (PAH) is a progressive syndrome with a poor prognosis. It is characterized by the marked remodeling of the pulmonary vasculature and a progressive rise in pulmonary vascular load, leading to right ventricular hypertrophy and remodeling [78]. The pathological features of PAH are the remodeling of the three layers of the distal pulmonary artery, which involves the uncontrolled growth of endothelial and smooth muscle cells and fibroblasts and infiltration by inflammatory cells, primarily into precapillary vessels [78], which results in luminal narrowing or the complete occlusion of small vessels. Currently, several therapeutic agents are available for the treatment of PAH. However, their effect on prognosis has not been satisfactory, and there is an urgent need to identify a novel therapeutic target and new drugs to act via that target. 

Previous studies showed that ANP modulates pulmonary vascular tone in rats with hypoxia-induced PAH [79,80]. Chronic intravenous infusion of ANP during chronic hypoxia slows the development of pulmonary hypertension and right ventricular enlargement [81], while the neutralization of endogenous ANP with a monoclonal antibody exacerbates hypoxia-induced PAH in adult rats [82]. This suggests that ANP exerts protective effects against pulmonary hypertension. BNP also exerts potent diuretic and systemic vasorelaxant effects via GC-A. An earlier study showed that BNP has a vasorelaxant effect on preconstricted thoracic aortic rings and pulmonary arterial rings, similar to that of ANP [83]. In addition, the chronic infusion of BNP reportedly prevents hypoxia-induced PAH more effectively than does ANP [84]. Collectively, these results suggest that both endogenous and exogenous ANP and BNP play an important role in preventing PAH. This hypothesis is supported by the observation that chronic hypoxia induces a pronounced and significantly greater increase in right ventricular hypertrophy and pressure in GC-A KO mice than in wild-type mice [85]. In addition, Klinger et al. showed that although CNP is a less potent vasodilator than ANP, chronic hypoxia increases plasma CNP levels [86].

Further investigations into the effectiveness of CNP in animal models of PAH have produced varying results. Itoh et al. reported that the continuous infusion of CNP ameliorated methacholine challenge-test-induced PAH in rats through regenerative and anti-apoptotic effects on endothelial cells, as well as anti-inflammatory and anti-fibrinolytic effects [87]. On the other hand, Casserly et al. reported that the continuous infusion of CNP did not affect the development of hypoxia-induced PAH [88]. In those two studies, rats were intravenously administered CNP in an identical fashion, using osmotic pumps. In addition, Nawa et al. confirmed that PAH was relieved by a constitutively active form of GC-B, which induced the massive simulation of cGMP synthesis, in a rat model where PAH was induced using the VEGF receptor inhibitor, SU5416, and hypoxia [89]. Thus, the effect of CNP may also depend on the degree of intensity of its action and the pathophysiology of pulmonary hypertension. A clear understanding of the role played by CNP in PAH will require further investigation.

## 11. Conclusions and Perspectives

**Figure 3:**
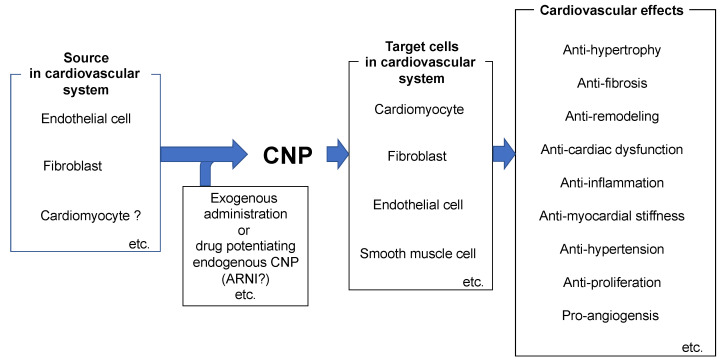
Significance of CNP in the cardiovascular system.

CNP was discovered to be the third natriuretic peptide, and its actions and biological significance have been studied in various ways. However, because they were discovered earlier, the status of the research and clinical applications of the ANP/ and BNP/GC-A systems are much beyond that of the CNP/GC-B system. Nevertheless, CNP has been shown not only to have similarities to ANP and BNP but also to have unique characteristics; its effects on the bone cartilage system have led to its clinical application in that context. In addition, with the recent advent of angiotensin receptor-neprilysin inhibitors (ARNI), which increase the endogenous levels of natriuretic peptides, the importance of natriuretic peptides, including CNP, is being reassessed (Figure 3). Therefore, we anticipate that further research into the roles of CNP in both normal physiological and pathophysiological processes will lead to its widespread application as a therapeutic and diagnostic agent, used in the treatment of cardiovascular diseases. 

## Data Availability

Not applicable.
